# Hyaluronic Acid Hampers the Inflammatory Response Elicited by Extracellular Vesicles from Activated Monocytes in Human Chondrocytes

**DOI:** 10.3390/pharmaceutics16111386

**Published:** 2024-10-28

**Authors:** Vittoria Carrabs, Maria Isabel Guillén, María Luisa Ferrándiz, María José Alcaraz, Fabio Ferrini, Rachele Agostini, Michele Guescini, Carmela Fimognari, Italo Capparucci, Elena Barbieri, Piero Sestili

**Affiliations:** 1 Dipartimento di Scienze Biomolecolari, University of Urbino Carlo Bo, 61029 Urbino, Italy; vittoria.carrabs@uchceu.es (V.C.); r.agostini4@campus.uniurb.it (R.A.); michele.guescini@uniurb.it (M.G.); italo.capparucci@uniurb.it (I.C.); elena.barbieri@uniurb.it (E.B.); 2 Instituto Interuniversitario de Investigación de Reconocimiento Molecular y Desarrollo Tecnológico (IDM), Universitat Politècnica de València, Universitat de València, 46010 Valencia, Spain; iguillen@uchceu.es (M.I.G.); luisa.ferrandiz@uv.es (M.L.F.); maria.j.alcaraz@uv.es (M.J.A.); 3 Departamento de Farmacia, Facultad de Ciencias de la Salud, Universidad CEU Cardenal Herrera, 46115 Valencia, Spain; 4 Dipartimento di Scienze per la Qualità della Vita, Università degli Studi di Bologna, C.so d’Augusto 237, 47921 Rimini, Italy; carmela.fimognari@unibo.it

**Keywords:** osteoarthritis, hyaluronic acid, extracellular vesicles, inflammation, infiltrative therapy, pathophysiology

## Abstract

**Background/Objectives:** Osteoarthritis (OA) is the most common joint disease in the adult population. OA is the result of multiple mechanisms leading to inflammation and the degradation of the cartilage. A complex series of etiological actors have been identified so far, including extracellular vesicles (EVs). The EV content of the synovial fluid (SF) can release inflammatory mediators that enhance OA progression. An intra-articular viscosupplementation of high-MW hyaluronic acid (HyA) constitutes the first-line conservative treatment for OA. Although attractive for the potential pharmacological implications, the possibility that HyA may interact with EVs in the context of OA has not yet been specifically investigated; therefore, the present study aimed to fill this gap. **Methods:** We studied the effect of a HyA preparation (a blend of crosslinked and linear polymers, CLHyA) on the relevant inflammatory markers in chondrocytes (HC cells or primary chondrocytes isolated from patients with advanced OA) exposed to the EVs collected from IL-1β-stimulated THP-1 human monocytes (EVs+). **Results:** EVs+ caused specific inflammatory responses in chondrocytes that could be prevented by coincubation with CLHyA. This anti-inflammatory activity is likely dependent on the direct binding of CLHyA to CD44 receptors highly expressed in EVs+ and on the subsequent hindrance to EVs+ diffusion and docking to target cells. **Conclusions:** On the whole, the tight interactions identified herein between HMW HyA and EVs+ represent a novel, pharmacologically exploitable mechanism potentially relevant in the context of OA treatment.

## 1. Introduction

Osteoarthritis (OA) is a prevalent joint disease affecting millions of adults globally, resulting in chronic inflammation and the degradation of the extracellular matrix [1], that more often develops in the knee joint, termed gonarthrosis (GA) [2]. The causes of the progressive joint degradation have been extensively studied; it is generally recognized that the degradation products themselves facilitate the progressive degeneration of cartilage through the receptor-mediated activation of chondrocytes and synoviocytes [3,4,5,6], giving rise to chronic inflammation [1]. Synoviocytes and chondrocytes are specialized in the synthesis, organization, and turnover of the components of the joint, namely collagen type II, aggrecans and hyaluronic acid (HyA).

HyA, the main component of the synovial fluid (SF), is a linear polysaccharide composed of thousands disaccharide monomers per molecule with a MW of 6–7 KDa, that lubricates and cushions the joint [7,8]. The molecular weight of HyA has a very high impact on OA; indeed, during OA and according to its progression, a significant decrease in both HyA concentration and mean MW can be observed [9,10].

Intra-articular HyA injections—termed viscosupplementation—to replenish the joint with HyA in the physiological range of MW are actually recognized as a safe and first-choice treatment for Kellgren II–III OA. The efficacy of HyA viscosupplementation is primarily attributed to its mechanical and viscosupplementation properties [11,12], but accumulating evidence suggests that the polymer is also capable of triggering protective biochemical pathways.

Injective formulations contain not only the high-MW linear polymer, but also the non-natural crosslinked form. The rationale of combining the crosslinked and the linear polymers is that the first—thanks to its higher resistance to hyaluronidases [13]—ensures a prolonged activity, while the second, substantially identical to the physiological HyA in SF, promotes more rapid beneficial effects [14,15,16,17,18]. The formulation used in this study (CLHyA) is representative of the many commercially available preparations and consists of a blend of high-MW linear and crosslinked hyaluronans [13].

OA is a multifactorial disease; evidence over the last decade has added another tile to this puzzling condition, showing that extracellular vesicles (EVs) such as exosomes and membranous EVs also contribute to the development and progression of the malady [19,20].

EVs transfer biologically active materials such as lipids, proteins, mRNA, miRNA, and DNA to target cells, modifying their condition [21,22]. Within joints, EVs can act in a bimodal pattern, either promoting or inhibiting inflammation [23,24,25]. In the OA-associated pro-inflammatory mode, EVs from IL-1β stimulated synovial fibroblasts significantly up-regulate matrix metalloprotease-13 (MMP-13) and ADAMTS-5 expression in articular chondrocytes, and down-regulate COL2A1 and aggrecan [26]; EVs accumulated within OA synovial fluid can also increase the production of several cytokines in M1-macrophages, resulting in a pro-inflammatory loop [27].

The aim of the present article is to investigate whether HyA (linear and/or crosslinked) can interact with EVs and modulate the EV-related cellular inflammatory responses in target chondrocytes. Indeed, the occurrence of interactions between HyA and EVs released in a pro-inflammatory environment might represent an additional druggable target paving the way to novel high-EV-affinity HyA formulations to ameliorate the management of low- to moderate-grade OA.

## 2. Materials and Methods

### 2.1. Chemicals

Unless otherwise stated, reagent grade chemicals, culture media, fetal bovine serum, and antibiotics were from Sigma Aldrich Europe (Darmstadt, Germany).

### 2.2. Isolation of Primary Chondrocytes

Primary chondrocytes (COA) were obtained from the knee cartilage samples of 15 patients with advanced OA (aged 50 and 79 years) subjected to total joint replacement. The experimental design was approved by the Institutional Ethical Committee (Comité Ético de Investigación en Humanos de la Comisión Ética en Investigación Experimental de la Universitat de València H1512058528966). The entire procedure was performed as per previously published protocol [28]. Small cartilage pieces were prepared from the femoral condyles and tibial plateau and minced in serum-free DMEM/Ham F-12 containing antibiotics (1%) overnight. To isolate COA, this preparation was digested enzymatically with 1 mg/mL type IA collagenase (Sigma–Aldrich, St. Louis, MO, USA) for 2 h in agitation at 37 °C. The digested tissue was filtered with a 70 μm nylon membrane (BD Biosciences Italy, Milano, Italy), washed, and resuspended in complete DMEM/HAM F-12. COA viability was assessed by the Trypan blue exclusion test. 

### 2.3. Cell Culture 

COA were cultured in DMEM/HAM F12 supplemented with antibiotics (100 units/mL penicillin, 100 µg/mL streptomycin) and FBS (10%). Commercially available human chondrocytes derived from healthy human articular cartilage (HC) were purchased from Lonza Biosciences (Visp, Switzerland), cultured in HC Growth medium (Lonza) and utilized for experiments between passage 5 to 10. All experiments were performed with COA or HC cultures at semi-confluence [28].

THP-1 cells (ATCC, Manassas, VA, USA) were routinely grown in RPMI 1640 supplemented with 10% heat-inactivated FBS and antibiotics (100 units/mL penicillin, 100 µg/mL streptomycin) and 2 mM L-glutamine. Cells were maintained in a humidified incubator at 37 °C with 5% CO_2_.

### 2.4. Isolation of EVs

THP-1 cells were resuspended in complete culture medium into two different culture flasks at a concentration of 1 × 10^6^ cells/mL, treated without or with 2 ng/mL IL-1β for 12 h. The cells were then centrifuged at 300× *g* 5 min, washed with complete medium, and resuspended at the same cellular concentration in serum free medium for a further 24 h. Cells were then centrifuged, the supernatant collected, and further centrifuged at 2000× *g* for 30 min at 4 °C; these supernatants were ultracentrifuged at 100,000× *g* at 4 °C for 90 min. After the removal of the supernatants, the pellets of EVs were stored a −80 °C. Two sets of EVs were obtained, namely EVs from unstimulated THP-1 cells (EVs−) and EVs from stimulated THP-1 (EVs+). For experiments, EVs− or EVs+ were resuspended in 50 μL of serum-free DMEM/HAM-F12 medium obtaining a concentration of 7.2 × 10^7^ particles/mL. This concentration was selected because it aligns with the concentration of EVs used by Guillén et al. in previous studies [29]. 

### 2.5. Treatment with CLHyA, EVs− or EVs+

COA or HC cells were seeded at a density of 1.5 × 10^5^ cells/well in 12-well plates and grown for 24 h before treatments, which were performed in a routine culture medium. At the semi-confluence stage, the cells were cultured with 0 or 0.3 mg/mL CLHyA (a mixture of 40% *W*/*W* 1 MDa, 40% 2 MDa BDDE-crosslinked HyA and 20% 500 KDa linear HyA) in the absence or presence of EVs−/EVs+ (7.2 × 10^7^ particles/mL) for 48 h or 7 days (in this latter case, at 72 h, the medium was replaced with fresh aliquots containing the same combinations of CLHyA and EVs−/EVs+) and then immediately assayed for the selected end-points. The experimental groups were control; 0.3 mg/mL CLHyA; 0.3 mg/mL CLHyA with EVs; and 0.3 mg/mL CLHyA with EVs+. 

The degree of HyA crosslinking has been assessed through the measurement of its extrusion force, as detailed in [30]; in particular, the extrusion force value of the BDDE-crosslinked HyA was 18 ± 1.2 Newton, compared to a value of 4.5 ± 0.38 Newton before crosslinking.

### 2.6. The Determination of Cell Viability with the MTT Assay

The mitochondrial-related conversion of 3-(4,5-dimethylthiazol-2-yl)-2,5 diphenyltetrazolium bromide (MTT) to reduced formazan was used to evaluate cell viability following exposure to EVs−, EVs+ or CLHyA. After treatments, cells were incubated with MTT (200 μg/mL) for 2 h; the medium was removed and cells were dissolved in DMSO (100 μL) to determine formazan formation at 550 nm by means of a Victor3V microplate reader (Perkin Elmer España, Madrid, Spain). Data were obtained from three to five separate experiments, each performed in duplicate.

### 2.7. Determination of IL-6 and MMP-13

The levels of IL-6 and MMP-13 were analyzed using ELISA kits. The sensitivities of the kits were 2.0 pg/mL for IL-6 (DuoSet ELISA kit, human; RnDSystems, Minnneapolis, MN, USA); 6.0 pg/mL for MMP-13 (Invitrogen, Waltham, MA, USA, Life Technologies, Carlsbad, CA, USA); and 28.125 pg/mL for COL-II (Wuhan Fine Biotech Co., Ltd., Wuhan, China). The assays were performed according to manufacturer’s recommendations. 

### 2.8. Characterization of EVs− and EVs+ from THP-1 Cells

EVs were analyzed by tunable resistive pulse sensing with a qNano instrument (IZON Sciences Ltd., Lyon, France) to determine their size distribution and concentration. Nanopore membranes (NP150) were utilized to measure the samples of EVs− and EVs+. At least 500 events/sample were scored. Calibration was performed using the reference bead CPC 200.

### 2.9. Quantification of Exosomal Markers in EVs and EVs+ 

The quantification of specific exosomal markers in EVs− and EVs+ samples was performed by ELISA kits. The following surface and cytosolic markers were assayed: CD9 of and CD81 as high sensitivity (100–1600 pg/mL) surface markers (Cell Guidance Systems, Cambridge, UK); TSG101 (Aviva Systems Biology, Corp., San Diego, CA, USA, sensitivity range of 23.44–1500 pg/mL), and PDCD6IP (Fine Biotech Co., Wuhan, China, sensitivity of 46.875 pg/mL). Human calnexin was assayed as negative control marker CNX (ABClonal, Düsseldorf, Germany), with a sensitivity of 0.17 ng/mL. Samples were analyzed using a a Victor3V microplate reader (PerkinElmer España, Madrid, Spain).

### 2.10. Internalization of EVs−/EVs+ in COA and HC Cells

EVs+ and/or EVs− internalization in COA or HC cells was assessed with fluorescent microscopy and its quantification in HC cells was assayed cytofluorimetrically. For experiments, EVs were prelabelled with PKH26 (System Biosciences, Palo Alto, CA, USA), which enables the tracking of their cellular uptake. COA or HC cells were then incubated with labeled EVs and visualized under a LEICA DM IL LED microscope equipped with a LEICA DFC425 C digital camera (Leica, Solms, Germany). The amount of EVs internalized by HC cells in the absence or presence of CLHyA was quantified cytofluorimeterically. Samples were acquired by a FACS Melody flow cytometer (Becton-Dickinson, Franklin Lakes, NJ, USA) and data were analyzed using FlowJo 10.5 software.

### 2.11. HyA or CLHyA Binding to EVs 

**Figure 1:**
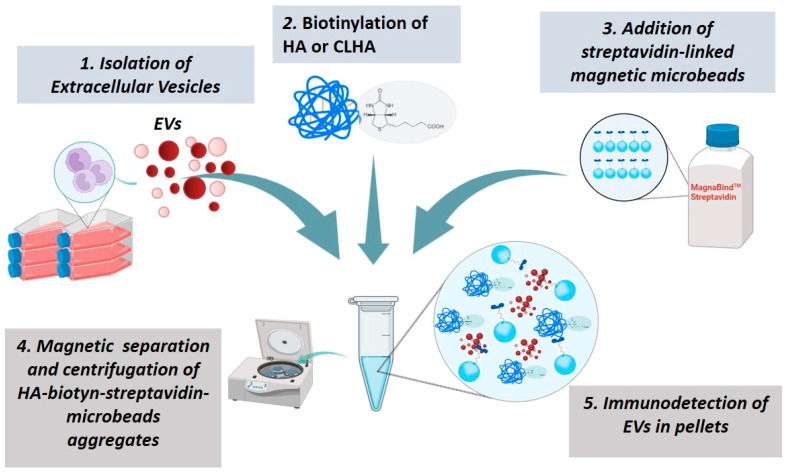
Visual scheme of the experimental approach to study HyA/CLHyA-EV interactions.

To determine whether hyaluronic acid binds to EVs+, biotinylated HyA or CLHyA was used. The approach consisted of the following five-step experimental procedure (see also Figure 1): (1) the biotinylation of CLHyA; (2) reaction between biotinylated CLHyA and EVs+ (3) reaction between CLHyA from step 2 with streptavidin-coated magnetic microbeads (4) the separation of magnetic microbeads (5) the identification of EVs+ in the microbead-separated fraction. 

Biotinylated 1 MDa linear HyA—included as a positive control—was obtained from Merck (Sigma-Aldrich Europe); biotynilated CLHyA was obtained using a biotinylation kit (Z-Link™ PFP-Biotin, Thermofisher, Monza, Italy) according to the manufacturer’s manual. 

Biotinylated -HyA or -CLHyA were incubated for 24 h with increasing concentrations of EVs− or EVs+ in serum-free culture medium. Streptavidin-linked magnetic microbeads (MagnaBind™ Streptavidin Beads, Thermofisher, Waltham, MA, USA) were added to the suspensions in order to allow the formation of biotin–streptavidin conjugates; the suspensions were then exposed to a magnetic field and centrifuged to separate and collect the magnetic microbeads; finally, the pellets were analyzed with Western blotting to assess the presence of EVs+ linked to magnetic microbeads; anti-TSG101 monoclonal antibodies (1:2000 dilution, clone 4A10 Abcam), an established marker of EVs, were used.

### 2.12. Western Blotting Analysis

For electrophoresis, samples were loaded onto 12% SDS-PAGE gels. Subsequently, proteins were blotted to a nitrocellulose membrane (GE Healthcare, Milan, Italy). The primary antibodies used were versus TSG101 (1:2000 dilution, clone 4A10 Abcam, Cambridge, UK) and CD44 (1:2000 dilution, clone HL1650 GeneTex). Primary antibodies were incubated overnight at 4 °C followed by washing and the application of a secondary HRP-conjugated antibody (Pierce, Thermofisher). Immune complexes were visualized using the Supersignal Dura reagent (Pierce, Thermofisher) and the obtained auto-radiographic films were quantified by ImageJ software V. 1.8.0 [31].

### 2.13. Statistical Analysis 

Data are expressed as the means ± SEM. Data were analyzed by one-way ANOVA followed by Tukey’s post-test using the GraphPad Prism 7.0 software (Graph Pad Software, La Jolla, CA, USA).

## 3. Results

### 3.1. Effect of CLHyA on the Viability and Inflammatory State of COA and HC Chondrocytes

**Figure 2:**
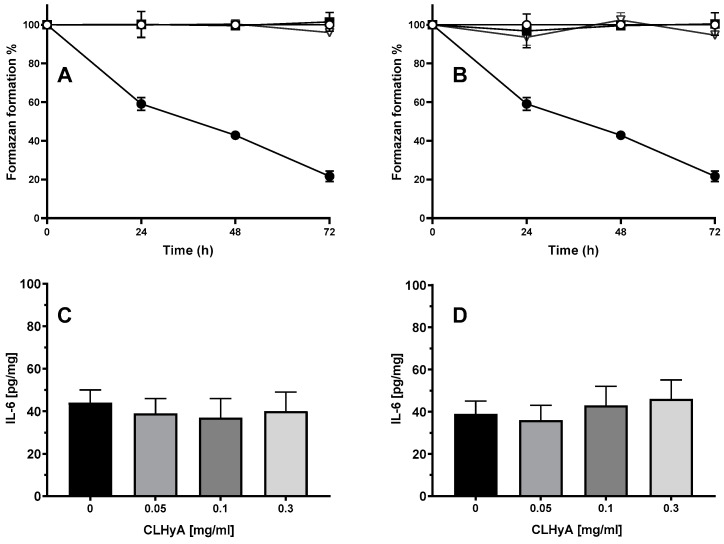
Cytotoxic and pro-inflammatory effect of CLHyA on COA or HC cells. COA (**A**,**C**) or HC (**B**,**D**) chondrocytes were treated with 0.05 (open circles), 0.1 (triangles), or 0.3 (squares) mg/mL CLHyA for 24, 48, or 72 h and then assayed for viability (**A**,**B**). Also shown is the effect of a 300 µM H_2_O_2_ challenge, included as a positive control. IL-6 levels in COA (**C**) or HC cells (**D**) treated with CLHyA for 48 h; values are the means ± S.E.M. of three independent experiments.

To determine the concentration range of CLHyA that does not exert any cytotoxic (MTT assay) or pro-inflammatory (the determination of the levels of IL-6 taken as an inflammation-like index) effect, COA or HC cells were exposed to a 0.05, 0.1, and 0.3 mg/mL of CLHyA: CLHyA per se did not cause any cytotoxic (Figure 2A) or pro-inflammatory effect in both cell lines (Figure 2B). Hence, the highest inactive concentration (0.3 mg/mL of CLHyA) was selected for the next experiments. 

### 3.2. Analysis of EVs from Unstimulated or Stimulated THP-1 Cell Line 

EVs were isolated from unstimulated (EVs−) or IL1β-stimulated (EVs+) THP-1 human monocytes. IL-1β was selected as a trigger to stimulate THP-1 since it is reportedly implicated in OA progression and known to promote the secretion of pro-inflammatory EVs in different cell types [32].

**Figure 3:**
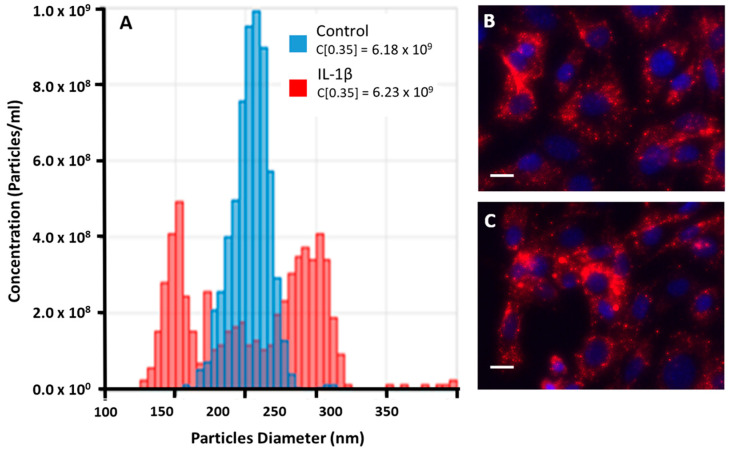
Size–distribution analysis of EVs. (**A**) Red and blue columns refer to EVs− and EVs+, respectively; particle concentrations are shown in the upper-left corner. (**B**,**C**) show EVs− and EVs+ extracellular vesicles (stained in red with PKH26) internalized within COA cells (nuclei stained with DAPI). Bars—10 µM.

The qNANO analyses showed that the net amount of EVs secreted by control or IL-1β -stimulated THP-1 cells was virtually identical (see Figure 3A); differently, size distribution analysis showed that EVs− piled up around a single peak of 200 nm while EVs+ gave rise to two distinct populations of 150 and 250 nm.

The phenotypic characterization showed that isolated EVs− and EVs+ were positive for CD9 (2.95 ± 0.31 and 2.35 ± 0.25 μg/mL, respectively) and CD81 (4.0 ± 0.39 and 5 ± 0.42 μg/mL), two specific transmembrane proteins of small EVs, as well as for PDCD6IP (0.103 ± 0.01 and 0.11 ± 0.012 pg/mL) and TSG101 (1.76 ± 0.19 and 2.36 ± 0.32 pg/mL), two specific cytosolic proteins of small EVs. Both EVs− and EVs+ were negative for CNX, a negative control, since it is peculiar of the membrane of the endoplasmic reticulum. 

### 3.3. EVs− and EVs+ Cellular Internalization 

The COA uptake study of EVs− and EVs+ (Figure 3B,C) showed that both were capable of crossing the cell membrane and being efficiently internalized within the cytoplasm, i.e., a condition reflecting the capacity of EVs− and EVs+ to interact with these primary cells; similarly, HC cells also internalized EVs− and EVs+ (see below). 

### 3.4. The Effect of CLHyA on the Levels of IL-6 and MMP-13 in Chondrocytes Exposed to EVs− or EVs+

**Figure 4:**
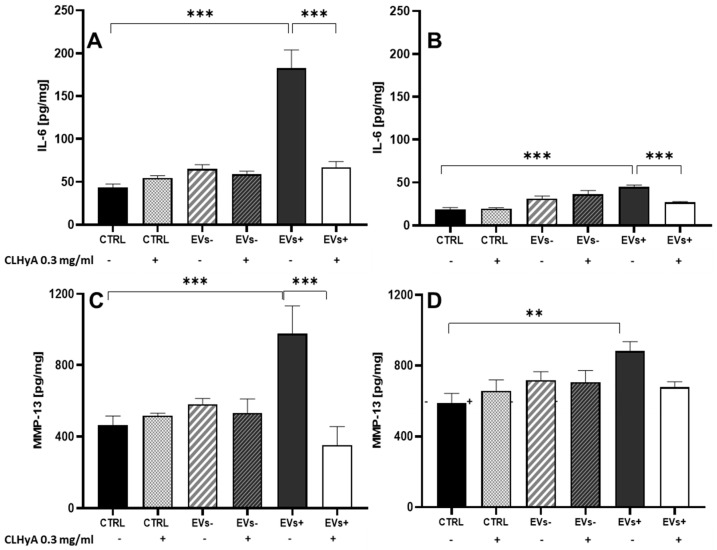
The evaluation of the levels of IL-6 and MMP-13. IL-6 (**A**,**B**) or MMP-13 (**C**,**D**) were evaluated in control, EVs− and EVs+ with or without 0.3 mg/mL CLHyA after 48 h (**A**,**C**) or 7 days (**B**,**D**) incubation. The levels of MMP-13 and of IL-6 were determined with an ELISA kit in the supernatants. Values are the means ± S.E.M. of five separate determinations (** *p* <0.01; *** *p* < 0.001).

The level of IL-6—a hallmark of the activation of cellular inflammatory events—released by COA cells in the culture medium were quantitated under the experimental conditions detailed in Figure 4. Exposure (48 h) to EVs alone or with 0.3 mg/mL CLHyA did not cause any increase in IL-6 levels. Exposure for the same incubation time to EVs+ caused a significant increase in IL-6 in COA cells and, interestingly, 0.3 mg/mL CLHyA nullified this response (Figure 4A). After 7 days of incubation (Figure 4B), the stimulatory effect of EVs+ was lower compared with the 48 h time period, although still significant compared to both control and EVs+/CLHyA-treated COA cells. The remaining exposure conditions illustrated in Figure 4A,B, namely Evs−, CLHyA and their combinations, were characterized by a slight, not significant trend toward increased IL-6 levels. 

The effect of EVs− and EVs+ with/without CLHyA on the levels of MMP-13 in COA cells is shown in Figure 4C,D. Interestingly, 48 h incubation with EVs+ resulted in a more than twofold increase in MMP-13 compared to control, an effect which was nullified when cells were co-incubated with CLHyA (Figure 4C). After 7 days (Figure 4D), a slight, although not significant increase in MMP-13 levels vs. control could still be observed in EVs+-treated cells; notably, CLHyA could prevent this effect. Qualitatively similar results for both IL-6 and MMP-13 levels were obtained in parallel experiments conducted on HC cells (see Supplementary Materials).

### 3.5. Interactions Between EVs+ and HyA 

To study the possible interactions between HyA and EVs+, a specific and articulated approach has been designed (see the Section 2 and Figure 1). This experimental design allows us (I) to assess the formation of chemical bonds/interactions between EVs+ and CLHyA; (II) to separate the resulting EVs+/CLHyA complexes from unbound EVs; (III) to demonstrate the presence of EVs hallmarks in the separated fraction.

**Figure 5:**
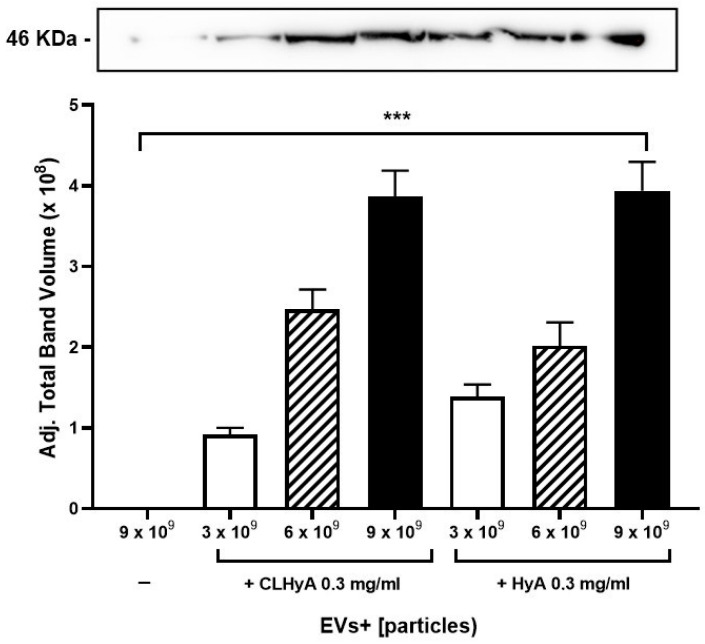
The determination of HyA-EV binding. A densitometric analysis of Western blots (four separate experiments; a representative Western blot is shown in the top of the panel) of TSG101 associated with microbeads bound to increasing amounts of EVs+ in the absence or presence of 0.3 mg/mL CLHyA or HyA. Data are the means ± S.E.M. from three separate determinations. *** *p*< 0.001.

**Figure 6:**
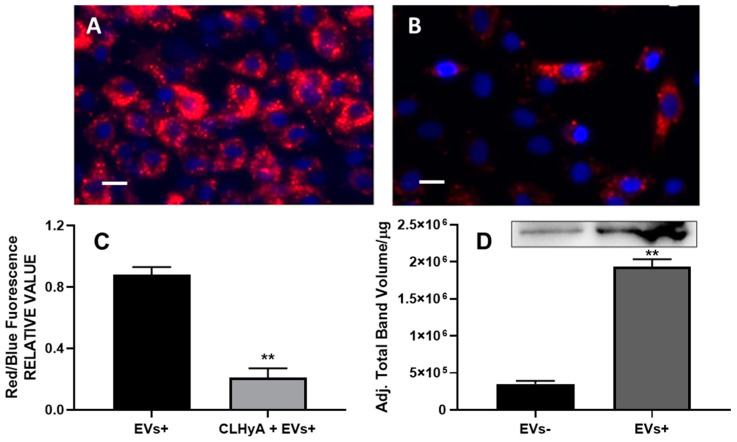
The cellular internalization of EVs+ in HC cells. Panels (**A**,**B**) representative micrographs of EVs+ internalization in HC cells in the absence (**A**) or presence (**B**) of 0.3 mg/mL CLHyA: red extracellular vesicles (stained with PKH26) surround blue HC cells’ nuclei (stained with DAPI); (**C**) the quantitative cytofluorimetric determination of EVs+ internalization in the absence or presence of 0.3 mg/mL CLHyA; (**D**) the expression level of CD44 in EVs− and EVs+ as assayed with Western blotting (the corresponding bands are shown in the inset). Bars—10 µm. ** *p* < 0.01.

The results of these experiments are shown in Figure 5 and indicate that a significant amount of the peculiar EV marker TSG101 could be found in the microbead-separated fraction; parallel determinations using pre-biotinylated commercially available HyA used as a positive control gave similar results. It is worth noting that, in both cases, TSG101 increased as a function of the amount of added EVs, a finding suggestive of a mass-dependent effect. A second set of experiments was carried out to see whether CLHyA could affect the entry of EVs+ into target cells. To this end, we incubated HC chondrocytes in the presence of EVs+ with or without CLHyA for 18 h, and then determined the extent of EVs+ accumulated within the cells. Figure 6A,B show two fluorescence micrographs representative of the above conditions; a quantitative analysis of the red/blue fluorescence confirmed that EVs accumulate within control cells, while in CLHyA-treated cells a significant reduction in EVs+ cellular internalization could be observed (Figure 6C). Quali-quantitatively similar results were obtained with cytofluorimetric analysis (see Supplementary Figures S1 and S2).

The results of the Western blotting of EVs+ and EVs− (Figure 6D) indicate that both EV populations express CD44 receptors, with EVs+ showing a significantly higher level compared to EVs−.

## 4. Discussion

The rationale of infiltrating OA joints with high-MW hyaluronans is based on multiple reasons, correlating with the pleiotropic nature of the polymer. The first one deals with the restoration of the mechanical properties of SF. In the course of active OA, the synthesis of HyA is reduced and, due to the concomitant increased activity of hyaluronidases, its mean MW progressively falls down, ending up far lower than in the SF of healthy subjects [32]. Under these conditions, the SF loses its viscoelastic/lubricating capacity, profoundly impacting on cartilage homeostasis and contributing to joint deterioration [11,12], an effect that can be counteracted by injecting high-MW HyA. Restoring the level of high-MW HyA has also been reported to limit the diffusion of pro-inflammatory cells (i.e., activated macrophages) within the joint [33]. 

In addition to the above mechanical—i.e., viscosupplementing—relevance, HyA has also been shown to exert other positive effects in OA at the biochemical level, such as A reduction in the levels of pro-inflammatory cytokines, binding, and interactions with CD44 receptors and nociceptors [34]. Considering the pleiotropic activity of HyA, we sought to investigate whether CLHyA might interfere/interact with EV signaling activity.

EVs released within SF play a bifaceted role, promoting trophic effects in healthy joints which turn detrimental over the course of active OA, where proteins and miRNAs delivered by EVs affect cell differentiation, cell survival, and joint inflammatory state [19,20]. The two populations of EVs utilized herein may be representative of these opposing natures, since they have been collected from IL-1β stimulated (EVs+) or mock-stimulated (EVs−) THP-1 promonocytoid cells. THP-1 are reportedly known to release EVs with distinct biological features depending on their state, namely inactive or pro-inflammatory [35]. 

Here, we obtained high amounts of EVs− and EVs+ from THP-1 cells, both possessing the biochemical hallmarks of genuine EVs but, importantly, different in their size distribution. Although the size distribution cannot be taken as a definitive indicator of biochemical and functional differences [35], it is plausible that the differences in size between EVs released by LPS-stimulated vs. unstimulated THP-1 cells mirrored their distinct biochemical and functional features. Similarly, we also found that continuous 48 h exposure (or 7 days in selected experiments) to the distinct EV types caused differential responses with regard to the levels of two etiologically relevant effectors, namely IL-6 and MMP-3. 

IL-6 is reportedly known to play a prominent role in OA pathology as well as OA pain [36,37], making IL-6 signaling an interesting target for OA treatment [38]; IL-6 levels in SF are elevated in OA patients and are 30- to 1000-fold higher in SF than in serum, indicative of the robust production and secretion by cells such as chondrocytes in the joint [39].

The matrix-degradative enzymes’ matrix metalloproteinases (MMPs) -1, -3 and -13, along with disintegrin and metalloproteinase with thrombospondin-1 domains (ADAMTS) -4 and -5, are involved at the etiologic level in the OA-associated destruction of the matrix proteins type II collagen, glycosaminoglycans, and proteoglycans [40,41,42]. Experiments with MMP-13-knockout mice have highlighted the importance of MMP-13 in OA pathogenesis; indeed, during OA progression, chondrocytes and synoviocytes release high levels of this cartilage-degrading enzyme [28,43]. Currently, MMP-13 is considered an attractive druggable target for clinically valuable pharmacological inhibitors [44].

Interestingly, both IL-6 and MMP-13 in COA and HC cells were significantly augmented upon EVs+ stimulation, while being unaffected by EVs−. The pro-inflammatory effect tended to decrease over time but could still be detected after 7 days of EVs+ exposure. 

Hence, in accordance with previous results from our group [28], EVs+ promote the accumulation of two biochemical effectors etiologically relevant to the progression of OA.

The second part of our experimental path was focused on ascertaining whether and how CLHyA (a formulation representative of the high-MW crosslinked-plus-linear HyA injective medical devices) could affect the responses evoked by EVs− or EVs+ on COA and HC cells. 

Importantly, we found that CLHyA prevented the robust increase in IL-6 and MMP-13 levels triggered by EVs+. 

Such an effect is conceivably related to the tight binding of CLHyA to EVs+, as demonstrated by the results of the microbead experiments. Indeed, this binding could result in the formation of CLHyA-EV supramolecular complexes unable to dock plasma membranes or enter target cells, and give rise to extracellular vesicles’ biological signaling (see also below). 

CD44 is an important adhesion molecule involved in cell–cell and cell–matrix interaction and in a large variety of cellular functions [45,46]; CD44 serves also as a cell surface receptor for HyA through a binding mechanism that involves the N-terminus of CD44 docking site lined by a mixture of primarily basic and hydrophobic amino acids [47]. In macrophages, CD44-HyA binding contributes to the adhesion to extracellular matrix, phagocytosis, migration and infiltration within inflammatory sites, and the secretion of cytokines [48]. CD44-positive monocytes increase their HyA-binding capacity upon activation by inflammatory stimuli. Interestingly, THP-1 monocytes have been shown to increase both CD44 membrane density and HyA binding capacity when subjected to pro-inflammatory stimuli such as phorbol–myristate–acetate or LPS [49]. 

Here, we found that CD44 is highly expressed in EVs+, probably as a remnant of the THP-1 plasma membrane. CD44 receptors on EVs may be responsible through the above mechanism for binding to CLHyA and for the ensuing preventive effects on the pro-inflammatory state of target chondrocytes. Once bound to extracellular vesicles, HyA or CLHyA could affect EVs+ activity in a bimodal pattern as follows: first, the polymers might coat EVs+ surface coating CD44 receptor via the above binding mechanism [47], thus hampering their docking and entry into COA or HCA chondrocytes, a condition impeding the release of EVs+ cargo to target cells; second, restoring the normal SF viscosity high-MW HyA and CLHyA might entrap EVs+, thus reducing their diffusion rate and frequency of interactions with target cells. 

Applying this notion to real-life healthy vs. pathological conditions, it can be hypothesized that in OA, the low viscosity of SF containing low-MW HyA allows for free EVs+ diffusion, cell targeting, and the delivery of the pro-inflammatory cargo; on the contrary, in high-MW hyaluronan-viscosupplemented joints, the restored viscosity would limit the EVs+ capacity to reach target cells and, finally, prevent the dissemination of inflammatory cascades. Interestingly, the viscosity of the surrounding medium has been shown to affect the movements of EVs within biological fluids, such as plasma [50]. 

## 5. Conclusions

To the best of our knowledge, this is the first report indicating that high-MW hyaluronans—either in the linear or crosslinked form—affect the cellular responses to the signals delivered by EVs+; this interaction might represent a new, additional mechanism responsible for the therapeutic activity of infiltrative hyaluronans in OA as well as a novel player in OA pathophysiology. Consequently, it can be inferred that this mechanism might operate in vivo through the reduction in EVs docking to—and uptake by—target cells, thus affecting their conditions, and that the EVs within OA SF might represent a druggable target. Future studies should be aimed at the identification of the fundamental structure–activity relationships (i.e., optimal MW, optimal type of polymer, e.g., linear vs. crosslinked or the optimal proportions of their blend) responsible for EVs-HyA interplay. The identification of such relationships could improve the control of pro-inflammatory EVs within OA joints.

In conclusion, further studies aimed at the characterization and comprehension of hyaluronic-acid–EVs+ reciprocal interactions under disease conditions may open the way to the rational development of more active preparations for the infiltrative treatment of OA.

## Data Availability

Data are contained within the article and Supplementary Materials.

## Supplementary Materials

The following supporting information can be downloaded at: https://www.mdpi.com/article/10.3390/pharmaceutics16111386/s1, Figure S1: Evaluation of the levels of IL 6 and MMP 13 in the HC cells IL 6 or MMP 13 were evaluated in control, EVs, and EVs with or without 0.3 mg/mL CLHyA after 48 h incubation. The amount of IL 6 and of MMP 13 was measured in the supernatants with an ELISA kit. Values represent the means S E M from three to five separate determinations (*** 0.001). Figure S2: Fluorescence-activated cell-sorting plots of EV internalization in HC cells with (or without (0.3 mg/mL CLHyA). The scatter blot with sequential gates (PE A PE–*PE A) for cell pools (A green, B red and control blue, HC cells) of one representative FACS experiment. The table summarizes the number of counted events and the total count of this representative experiment; Figure S3: Fluorescence micrograph of HC cells only incubated with DAPI (blue) and PKH26 (red); Figure S4: Uncropped ex Figure 5.

## References

[1] Kim H.A. (2022). Osteoarthritis-Insights from recent research. J. Rheum. Dis..

[2] Ashford S., Williard J. (2014). Osteoarthritis: A review. Nurse Pract..

[3] Werb Z., Tremble P.M., Behrendtsen O., Crowley E., Damsky C.H. (1989). Signal transduction through the fibronectin receptor induces collagenase and stromelysin gene expression. J. Cell Biol..

[4] Homandberg G.A., Meyers R., Xie D.L. (1992). Fibronectin fragments cause chondrolysis of bovine articular cartilage slices in culture. J. Biol. Chem..

[5] Yasuda T., Poole A.R. (2002). A fibronectin fragment induces type II collagen degradation by collagenase through an interleukin-1-mediated pathway. Arthritis Rheum..

[6] Yasuda T., Poole A.R., Shimizu M., Nakagawa T., Julovi S.M., Tamamura H., Fujii N., Nakamura T. (2003). Involvement of CD44 in induction of matrix metalloproteinases by a COOH-terminal heparin-binding fragment of fibronectin in human articular cartilage in culture. Arthritis Rheum..

[7] Volpi N., Schiller J., Stern R., Soltes L. (2009). Role, Metabolism, Chemical Modifications and Applications of Hyaluronan. Curr. Med. Chem..

[8] Fraser J.R., Laurent T.C., Laurent U.B. (1997). Hyaluronan: Its nature, distribution, functions and turnover. J. Intern. Med..

[9] Knopf-Marques H., Pravda M., Wolfova L., Velebny V., Schaaf P., Vrana N.E., Lavalle P. (2016). Hyaluronic Acid and Its Derivatives in Coating and Delivery Systems: Applications in Tissue Engineering, Regenerative Medicine and Immunomodulation. Adv. Healthc. Mater..

[10] Ebid R., Lichtnekert J., Anders H.J. (2014). Hyaluronan Is Not a Ligand but a Regulator of Toll-Like Receptor Signaling in Mesangial Cells: Role of Extracellular Matrix in Innate Immunity. ISRN Nephrol..

[11] Maheu E., Bannuru R.R., Herrero-Beaumont G., Allali F., Bard H., Migliore A. (2019). Why we should definitely include intra-articular hyaluronic acid as a therapeutic option in the management of knee osteoarthritis: Results of an extensive critical literature review. Semin. Arthritis Rheum..

[12] Migliore A., Frediani B., Gigliucci G., Anichini S.E., Cassol M., Crimaldi S., De Lucia O., Iolascon G., Foti C. (2018). One-year follow-up showing effects of single intra-articular injection of hyaluronic acid (1,500–2,000 kDa) in symptomatic knee osteoarthritis. J. Biol. Regul. Homeost. Agents..

[13] Barbieri E., Capparucci I., Mannello F., Annibalini G., Contarelli S., Vallorani L., Gioacchini A.M., Ligi D., Maniscalco R., Gervasi M. (2019). Efficacy of a Treatment for Gonarthrosis Based on the Sequential Intra-Articular Injection of Linear and Cross-Linked Hyaluronic Acids. Muscle Ligaments Tendons J..

[14] Kreger S.T., Voytik-Harbin S.L. (2009). Hyaluronan concentration within a 3D collagen matrix modulates matrix viscoelasticity, but not fibroblast response. Matrix Biol..

[15] Kawasaki K., Ochi M., Uchio Y., Adachi N., Matsusaki M. (1999). Hyaluronic acid enhances proliferation and chondroitin sulfate synthesis in cultured chondrocytes embedded in collagen gels. J. Cell. Physiol..

[16] Frean S.P., Abraham L.A., Lees P. (1999). In vitro stimulation of equine articular cartilage proteoglycan synthesis by hyaluronan and carprofen. Res. Vet. Sci..

[17] Takahashi K., Goomer R.S., Harwood F., Kubo T., Hirasawa Y., Amiel D. (1999). The effects of hyaluronan on matrix metalloproteinase-3 (MMP-3), interleukin-1β(IL-1β), and tissue inhibitor of metalloproteinase-1 (TIMP-1) gene expression during the development of osteoarthritis. Osteoarthr. Cartil..

[18] Goto M., Hanyu T., Yoshio T., Matsuno H., Shimizu M., Murata N., Shiozawa S., Matsubara T., Yamana S., Matsuda T. (2001). Intra-articular injection of hyaluronate (SI-6601D) improves joint pain and synovial fluid prostaglandin E2 levels in rheumatoid arthritis: A multicenter clinical trial. Clin. Exp. Rheumatol..

[19] Withrow J., Murphy C., Liu Y., Hunter M., Fulzele S., Hamrick M.W. (2016). Extracellular vesicles in the pathogenesis of rheumatoid arthritis and osteoarthritis. Arthritis Res. Ther..

[20] Xie F., Liu Y., Chen X., Li Q., Zhong J., Dai B., Shao X., Wu G. (2020). Role of MicroRNA, LncRNA, and Exosomes in the Progression of Osteoarthritis: A Review of Recent Literature. Orthop. Surg..

[21] Gibbings D.J., Ciaudo C., Erhardt M., Voinnet O. (2009). Multivesicular bodies associate with components of miRNA effector complexes and modulate miRNA activity. Nat. Cell. Biol..

[22] Robbins P.D., Dorronsoro A., Booker C.N. (2016). Regulation of chronic inflammatory and immune processes by extracellular vesicles. J. Clin. Investig..

[23] Alcaraz M.J., Compañ A., Guillén M.I. (2019). Extracellular Vesicles from Mesenchymal Stem Cells as Novel Treatments for Musculoskeletal Diseases. Cells.

[24] Alcaraz M.J., Guillén M.I., Ferrándiz M.L. (2019). Emerging therapeutic agents in osteoarthritis. Biochem. Pharmacol..

[25] Wu W.C., Song S.J., Zhang Y., Li X. (2020). Role of Extracellular Vesicles in Autoimmune Pathogenesis. Front. Immunol..

[26] Kato T., Miyaki S., Ishitobi H., Nakamura Y., Nakasa T., Lotz M.K., Ochi M. (2014). Exosomes from IL-1β stimulated synovial fibroblasts induce osteoarthritic changes in articular chondrocytes. Arthritis Res. Ther..

[27] Domenis R., Zanutel R., Caponnetto F., Toffoletto B., Cifù A., Pistis C., Di Benedetto P., Causero A., Pozzi M., Bassini F. (2017). Characterization of the Proinflammatory Profile of Synovial Fluid-Derived Exosomes of Patients with Osteoarthritis. Mediat. Inflamm..

[28] Carrabs V. (2023). Role of Microvesicles in Gonarthrosis and Their Modulation by Hyaluronic Acid Administered in Viscosupplementation for the Rational Development of Innovative Medical Devices. Ph.D. Thesis.

[29] Guillén M.I., Tofiño-Vian M., Silvestre A., Castejón M.A., Alcaraz M.J. (2021). Role of peroxiredoxin 6 in the chondroprotective effects of microvesicles from human adipose tissue-derived mesenchymal stem cells. J. Orthop. Translat..

[30] Tezel A., Fredrickson G.H. (2014). The science of hyaluronic acid dermal fillers. J. Cosmet. Laser Ther..

[31] Guescini M., Leo G., Genedani S., Carone C., Pederzoli F., Ciruela F., Guidolin D., Stocchi V., Mantuano M., Borroto-Escuela D. (2012). Microvesicle and tunneling nanotube mediated intercellular transfer of g-protein coupled receptors in cell cultures. Exp. Cell. Res..

[32] Necas J., Bartosikova L., Brauner P., Kolar J. (2008). Hyaluronic acid (hyaluronan): A review. Vet. Med..

[33] Rayahin J.E., Buhrman J.S., Zhang Y., Koh T.J., Gemeinhart R.A. (2015). High and Low Molecular Weight Hyaluronic Acid Differentially Influence Macrophage Activation. ACS Biomater. Sci. Eng..

[34] Michalczyk M., Humeniuk E., Adamczuk G., Korga-Plewko A. (2022). Hyaluronic Acid as a Modern Approach in Anticancer Therapy-Review. Int. J. Mol. Sci..

[35] Wang J.G., Williams J.C., Davis B.K., Jacobson K., Doerschuk C.M., Ting J.P.Y., Mackman N. (2011). Monocytic microparticles activate endothelial cells in an IL-1β–dependent manner. Blood.

[36] Eitner A., Hofmann G.O., Schaible H.G. (2017). Mechanisms of Osteoarthritic Pain. Studies in Humans and Experimental Models. Front. Mol. Neurosci..

[37] Wiegertjes R., van de Loo F.A.J., Blaney Davidson E.N. (2020). A roadmap to target interleukin-6 in osteoarthritis. Rheumatology.

[38] Eitner A., König C., Kohler F.C., Hofmann G.O., Wildemann B., Aurich M., Schaible H.-G. (2024). Importance of IL-6 trans-signaling and high autocrine IL-6 production in human osteoarthritic chondrocyte metabolism. Osteoarthr. Cartil..

[39] Tsuchida A.I., Beekhuizen M., Rutgers M., van Osch G.J., Bekkers J.E., Bot A.G., Geurts B., Dhert W.J., Saris D.B., Creemers L.B. (2012). Interleukin-6 is elevated in synovial fluid of patients with focal cartilage defects and stimulates cartilage matrix production in an in vitro regeneration model. Arthritis Res. Ther..

[40] Tchetina E.V., Squires G., Poole A.R. (2005). Increased type II collagen degradation and very early focal cartilage degeneration is associated with upregulation of chondrocyte differentiation related genes in early human articular cartilage lesions. J. Rheumatol..

[41] Michael J.W.P., Schlüter-Brust K.U., Eysel P. (2010). The Epidemiology, Etiology, Diagnosis, and Treatment of Osteoarthritis of the Knee. Dtsch. Arztebl. Int..

[42] Wu C.W., Tchetina E.V., Mwale F., Hasty K., Pidoux I., Reiner A., Geurts B., Dhert W.J., Saris D.B., Creemers L.B. (2002). Proteolysis Involving Matrix Metalloproteinase 13 (Collagenase-3) Is Required for Chondrocyte Differentiation That Is Associated with Matrix Mineralization. J. Bone Miner. Res..

[43] Takaishi H., Kimura T., Dalal S., Okada Y., D’Armiento J. (2008). Joint Diseases and Matrix Metalloproteinases: A Role for MMP-13. Curr. Pharm. Biotechnol..

[44] Fuerst R., Choi J.Y., Knapinska A.M., Cameron M.D., Ruiz C., Delmas A., Sundrud M.S., Fields G.B., Roush W.R. (2022). Development of a putative Zn2+-chelating but highly selective MMP-13 inhibitor. Bioorg. Med. Chem. Lett..

[45] Naor D., Sionov R.V., Ish-Shalom D. (1997). CD44: Structure, function, and association with the malignant process. Adv. Cancer Res..

[46] Stamenkovic I., Aruffo A., Amiot M., Seed B. (1991). The hematopoietic and epithelial forms of CD44 are distinct polypeptides with different adhesion potentials for hyaluronate-bearing cells. EMBO J..

[47] Bhattacharya D., Svechkarev D., Souchek J.J., Hill T.K., Taylor M.A., Natarajan A., Mohs A.M. (2017). Impact of structurally modifying hyaluronic acid on CD44 interaction. J. Mater. Chem. B.

[48] Kobayashi T., Chanmee T., Itano N. (2020). Hyaluronan: Metabolism and Function. Biomolecules.

[49] Gee K., Lim W., Ma W., Nandan D., Diaz-Mitoma F., Kozlowski M., Kumar A. (2002). Differential Regulation of CD44 Expression by Lipopolysaccharide (LPS) and TNF-α in Human Monocytic Cells: Distinct Involvement of c-Jun N-Terminal Kinase in LPS-Induced CD44 Expression. J. Immunol..

[50] Božič D., Sitar S., Junkar I., Štukelj R., Pajnič M., Žagar E., Kralj-Iglič V., Kogej K. (2019). Viscosity of Plasma as a Key Factor in Assessment of Extracellular Vesicles by Light Scattering. Cells.

