# Supplementary material for: Hyaluronic Acid Hampers the Inflammatory Response Elicited by Extracellular Vesicles from Activated Monocytes in Human Chondrocytes
Source: Pharmaceutics. 2024 Oct 28;16(11):1386. doi: 10.3390/pharmaceutics16111386 (PMC11597363; doi:10.3390/pharmaceutics16111386)
Supplement: Supplementary file 1 [file pharmaceutics-16-01386-s001.zip › pharmaceutics-3207605-supplementary.pdf]

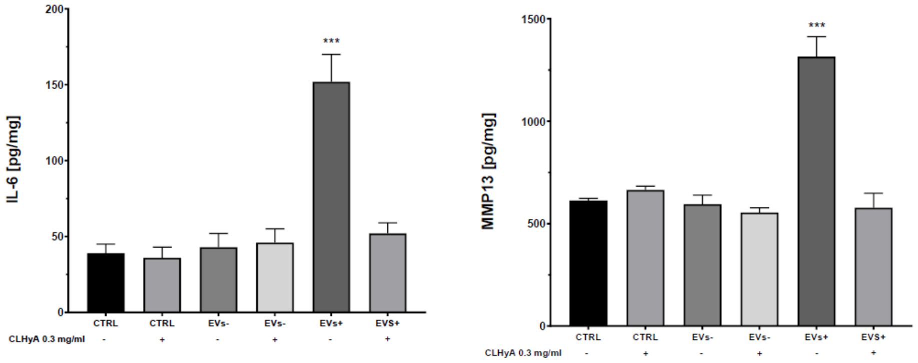

Figure S1. Evaluation of the levels of IL-6 and MMP-13 in HC cells.IL-6 or MMP13 were evaluated in control, Evs and EVs+ with or without 0.3mg/ml CLHyA after 48h incubation. The amount of IL 6 and of MMP13 was measured in the supernatants with an ELISA kit. Values represent the means+S.E.M. from three to five separate determinations (***p<0.001).

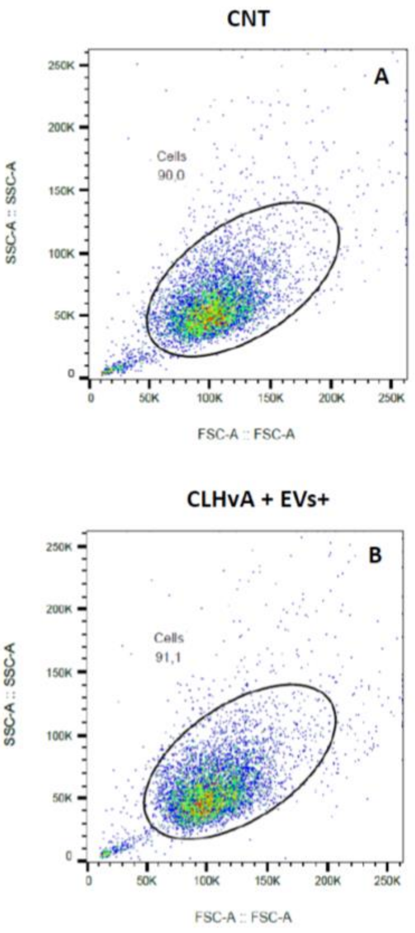

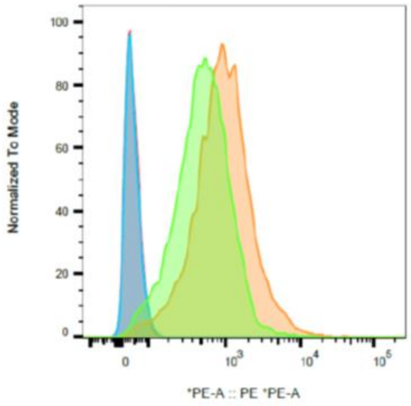

	Sample Name	Count	Mean: *PE-A
	Ctrl	5831	48,1
	CLHyA 0.3 mg/ml	5777	48,7
	EVs+	5865	1454
	EVs+ + CLHyA 0.3 mg/ml	5825	758

Figure S2. Fluorescence activated cell sorting plots of EVs+ internalization in HC cells with (B) or without (A) 0.3mg/ml CLHyA.Shown is the scatter blot with sequential gates (PE-A::PE-*PE-A) for cell pools (A green, B red and control blue,HC cells) of one representative FACS experiment. The table summarizes the number of counted events and of the total count of this representative experiment.

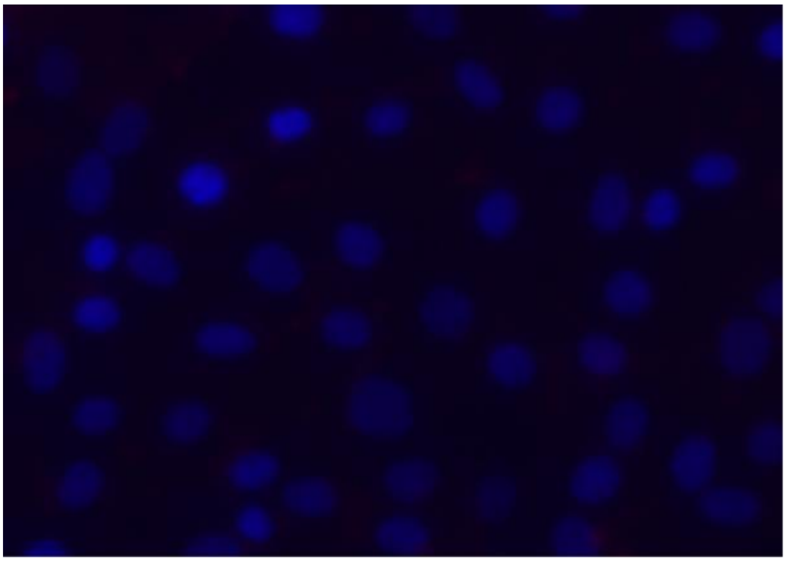

Figure S3. Fluorescence micrograph of HC cells only incubated with DAPI (blue) and PKH26 (red).

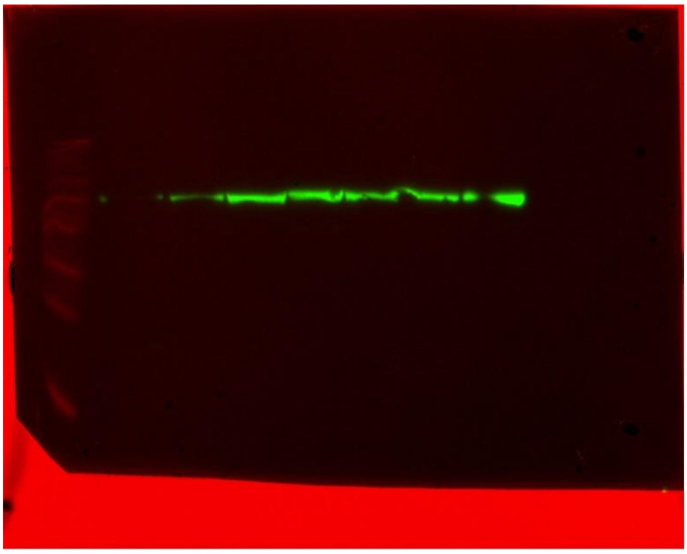

Figure S4 Uncropped ex Figure 5.
